# Demographic, endoscopic and histological profile of esophageal cancer at the Gastroenterology Department of Maputo Central Hospital from January 2016 to December 2018

**DOI:** 10.11604/pamj.2022.41.100.30941

**Published:** 2022-02-04

**Authors:** Muhammad Ismail, Liana Mondlane, Michella Loforte, Luzmira Dimande, Sheila Machatine, Carla Carrilho, Jahit Sacarlal

**Affiliations:** 1 Serviço de Gastroenterologia, Hospital Central de Maputo, Maputo, Mozambique,; 2 Serviço de Anatomia Patológica, Hospital Central de Maputo, Maputo, Mozambique,; 3 Departamento de Patologia, Faculdade de Medicina, UEM, Maputo, Mozambique

**Keywords:** Mozambique, esophageal neoplasms, carcinoma, endoscopy, gastrointestinal, public health

## Abstract

**Introduction:**

esophageal cancer is a major public health problem in Mozambique. It is the nineth most common cancer worldwide in terms of incidence (604.000 new cases/year), and sixth in overall mortality (544.076 deaths/year). In Mozambique esophageal cancer was the seventh most common cancer in males and the fifth in females between 1991 and 2008.

**Methods:**

it was done a cross-sectional hospital-based epidemiological study, using secondary demographics endoscopic and pathologic features data. A retrospective analysis of the existing information of patients classified as esophageal cancer diagnosed with upper gastrointestinal endoscopy observed from January 1^st^, 2016 to December 31^st^, 2018 at the Gastroenterology Service of Maputo Central Hospital. A coding sheet was created a priori, and data analysed in SPSS version 20.

**Results:**

of the 205 cases with complete records where included in the analysis, there was a higher frequency of females with 56.6% (116/205). The average age was 59.5 years with standard deviation of ± 12.9 years. Most of the patients were native of southern Mozambique, with 92.7% (190/205), of which Maputo made up 53.2% (109/205). Regarding race, 99.5% (204/205) were black. The most affected endoscopic location was the middle third with 48.8% (100/205), followed by the lower third with 29.8% (61/205) and the upper third with 21.5% (44/205). Squamous cell carcinoma was the most frequent, with 92.7% (190/205), followed by adenocarcinoma with 4.9% (10/205).

**Conclusion:**

due to the high number of observed cases of esophageal cancer, a high degree of clinical suspicion is needed for timely diagnosis and more effective treatment. Updated prevalent studies are needed throughout the country to understand the true impact of esophageal cancer on the Mozambican population.

## Introduction

The esophagus is a muscular tube which links the pharynx to the stomach, and has the main function for driving food from the mouth to the stomach. This organ in adults measures approximately 25 cm to 30 cm and can vary accordingly with the individual height [1]. Can be divided in cervical (superior/upper), thoracic (medium/middle) and abdominal (inferior/lower) esophagus. Endoscopically this division is measured by the distance in centimeters of the incisive teeth with the endoscopy [2]. Esophageal cancer (EC) is one of the most deadly in the world due to its nature that is rather aggressive and with low survival rate [3]. It is the nineth most common cancer in the world in terms of incidence (604.100 new cases/year), and the sixth in general mortality (544.076 death/year) [4], being more than 80% of the total cases and death occurred in underdeveloped countries [5]. The incidence has been increasing over the years [6]. In general, the incidence rate is twice higher in geographic underdeveloped regions in relation to developed countries [7]. The major incidence of the EC can be found in the region denominated “esophageal cancer belt” which is extended from North - Center of China to Iran and from the East to the South of Africa, being the countries with the major incidence since the past time [8]. In Sub-Saharan Africa, there are many geographic variabilities, with a major incidence of EC reported in Malawi with 30.3/100.000 (cases/year) in males and 19.4/100.000 (cases/years) in females [9]. In Mozambique between 2015-2017 EC was the fourth most cancer in males and fifth in female. The incidence rates of this cancer are relatively higher for the international standards [10]. Most of the patients in our setting is diagnosed at advanced state of the disease, with weak nutritional states, which makes the treatment difficult. The median survival time is 3.5 months for all patients, and 8.7 months for patients treated with curative intent [11]. The male to female ratio for EC is of 2 to 5 : 1, and the incidence increases in people in the age group of over 50 years old [12]. In the Western World, the most affected age group is between 60 to 90 years [13], meanwhile, in Africa (Malawi, Mozambique, Uganda and Kenya) it is more common between 40 to 59 years old [14].

The two most important histologic types are, squamous cell carcinoma (SCC) and adenocarcinoma (ADC) [15], however some other rare types may be mentioned. The SCC histologic type is more frequent, but this tendency is changing in the last years, in favour of ADC, mainly in the developed countries [16]. In Africa the SCC is still by far the most frequent histologic type [17]. In a recent study carried out in Mozambique, SCC corresponded to 94.4% (369/391 cases) of all esophageal carcinomas [11]. The cancer location inside the esophagus length varies with histologic type [18], being the SCC, mainly found in the medium and the distal third of esophagus and the ADC more commonly located in the distal third [19].

The risk factors for the two subtypes of EC vary. Various factors are implicated such as gender, race, habits and lifestyles and some pre-conditions. The SCC is more frequent in black males and white females, while the ADC is more frequent in white males. The incidence of ADC is higher in males and white males. Tobacco is the focal risk factor contributing to the development of the SCC. According to verified data, individuals who have stopped smoking for a period of 10 years have a higher risk of developing EC than individuals who have never smoked. The amount of cigarettes smoked is also an important factor to be taken into consideration. Alcohol is another risk factor, varying according to the amount ingested. Ethanol is a substance metabolized by alcohol dehydrogenase to form acetaldehyde. Acetaldehyde, in turn, in contact with the mucous membrane, induces DNA mutation and promotes the development of cancer. Foods rich in nitrogen compounds are related to the increased incidence of SCC. Individuals with a vitamin and mineral deficiency due to low intake of vegetables and fruits are a target group at risk of developing SCC [20, 21].

Gastro-oesophageal reflux disease (GERD) can cause ADC either directly, or through the formation of a pre-neoplastic lesion, Barret's esophagus (BE). Barret's esophagus is metaplasia, which occurs in 6%-14% of patients with GERD, and of which approximately 0.5%-1% have the possibility of developing ADC. Obesity is the largest and most consistent risk factor for the development of ADC. There are two main mechanisms by which increased weight influences the development of ADC. First is the physical mechanism, which in turn increases the incidence of GERD, and second is the hormone-dependent mechanism, mediated by inflammatory markers that are secreted by adipocytes [20, 21].

The EC is usually asymptomatic in the initial stages. In an advanced stage, the patients can show symptoms of progressive dysphagia, non-intentional loss of weight (10% or more), odynophagia, recent dyspepsia, pirosis, pain in the chest or upper gastrointestinal bleeding. In these symptoms, the isolated dysphagia or combined with the non-intentional loss of weight is a very common symptom in patients with EC [22]. The patient with suggestive symptoms such as dysphagia, upper gastrointestinal bleeding, recurrent aspiration pneumonia, vomiting and weight loss should be submitted to upper gastrointestinal endoscopy (UGE), with biopsies if a lesion suggestive of cancer is found [23, 24]. The decision about the initial approach of EC treatment are taken based on the clinical and pathological stage [23], which should be done with a high level of precision [25, 26]. In addition to the endoscopic study required for diagnosis, other imaging methods are required for staging such as esophageal barium radiography, echoendoscopy, computed tomography (CT), positron emission tomography (PET-CT) and magnetic resonance imaging (MRI). The initial staging test is the thoracic-abdominal CT, including the supraclavicular region. According to availability, it can be replaced by a PET-CT. The advantage of PET-CT is the identification of metastasis not detected by other techniques. In cases of early disease, echoendoscopy is indicated for better staging. The use of MRI is an alternative that, although not used systematically, plays a complementary role, covering the limitations of other techniques. Barium radiography of the esophagus has already been used in the past as an initial diagnostic method, but nowadays it is important in cases of stenosing tumors that impede the progression of the endoscope, to evaluate the location and extent of the lesion [27]. The esophagus is a muscular tube that connects the pharynx to the stomach, the wall of which is formed by four layers: mucosa, submucosa, muscularis propria and adventitia. Because it does not have a serosa, in the presence of a neoplasm there is a rapid invasion of cancer into neighbouring structures of the neck and mediastinum [27]. Unfortunately, most patients with EC when they go to the health unit show local tumor invasion or metastasis to other organs, and are no longer amenable to curative treatment [28]. In early cancer, endoscopic treatment is the treatment of choice, trough endoscopic mucosal and submucosal dissection [26]. For locally advanced EC (cT3-T4 or cN1-3 M0), neoadjuvant chemotherapy is performed on a cisplatin and 5-fluorouracil basis and an esophagectomy [29, 30]. In the case of advanced unresectable tumors (M1), the treatment is palliative, in order to improve the nutritional status, control dysphagia, improve quality of life and prolong survival. There are several types of palliative treatment, including chemotherapy, placement of self-expanding esophageal prosthesis, brachytherapy, percutaneous endoscopic gastrostomy and others [31-33]. Surgical resection associated with adjuvant chemotherapy or chemoradiotherapy is curative in approximately 50% of patients with the operable disease (cT3-T4 or cN1-3 M0), but is also associated with significant morbidity. Therefore, precise preoperative staging is necessary to spare patients unnecessary toxicity and futile surgery [34]. The treatment of EC is a challenge and requires a multidisciplinary approach to improve outcomes. The results of additional and ongoing clinical trials will help to establish the most appropriate interdisciplinary strategy for each stage of each histological subtype [35]. EC is a weakening pathology with a higher rate of morbi-mortality. In the diagnosed cases, the major cases have the criteria for relieving treatment, resulting, invariably in individual morbidity and mortality in a short term period [3].

Strategies for EC screening targeted at early diagnosis can improve prognosis. There are few studies on the demographic and pathological profile of EC in Mozambique [11], and it is extremely important to study the epidemiological profile of this nosological entity in order to increase its early detection programmes [36] and also its prevention [3]. The aim of our study was to describe the demographic, endoscopic and pathologic features of patients with EC diagnosed at the Gastroenterology Department at Maputo Central Hospital (MCH) in Mozambique.

## Methods

**Study design and setting:** a cross-sectional, hospital-based study was carried out using secondary data, focusing on demographic, endoscopic and histological findings. A retrospective data analysis was carried out at the Gastroenterology service of MCH from 1^st^ January 2016 to 31^st^ December 2018 on patients classified as EC diagnosed by UGE. The MCH is a teaching hospital, of quaternary level and of national reference, being the largest in the country. It is a hospital with more than 100 years of existence, with 1500 beds, that directly assists about 2,000,000 inhabitants in the city and province of Maputo.

**Participants:** the study included data of all patients submitted to the UGE in the gastroenterology service of MCH from January 2016 to December 2018. A suggestive lesion of EC was detected, with subsequent endoscopic biopsy. The patients excluded were those whose endoscopy reports were incomplete concerning the demographic and/or endoscopic data, the histological report was unfinished, with a lack of information or who did not show any feature of cancer histologically.

**Variables and data sources:** the data were collected using a printed report for collecting data and included demographic characteristics, endoscopic topography, histologic characteristics and the relation between the histologic diagnosis and the endoscopic topography of cancer. The data were organised and analysed through Statistical Package for the Social Sciences SPSS Statistics Data, version 20. The description of demographic profile, endoscopic and histologic, were summed up in forms of frequency (average, median and mode). The correlation between the histologic diagnosis and endoscopic topography of the cancer was carried out through a test of “Chi-square” assuming a level of significance of 5%.

**Figure 1:**
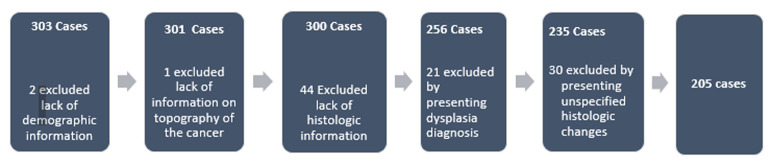
patient recruitment flowchart

**Study size:** from 303 cases (suspected cases of EC through UGE), 2 of them were excluded from the study due to the lack of demographic information and 1 case due to the lack of information about the endoscopic location of cancer. There were also excluded 44 cases due to the lack of histologic information of cancer because of information system failure in the pathologic anatomy unity at the MCH. Twenty-one (21) cases were excluded, as they presented suggestive histologic founds of dysplasia (moderate to high), and 30 by presenting unspecified histologic changes. The remaining of 205 cases were used for carrying out this study (Figure 1).

**Ethical considerations:** the study was approved by the Institutional Committee on Bioethics for Health (reference No. CIBS FM & HCM/37/2019).

## Results

There has been a major frequency of the cancer in females with 56.6%. When distributed by age groups, it is noticed as a major frequency in the 5^th^ and 6^th^ decade of life, with 33.7% in the age group of 51 to 60 years old, and a lower frequency in patients with less than 30 years, corresponding to 1.5%. The age in which the cancer was more frequent was 60 years, the minimum age reported was 30 years and the maximum of 97 years. The average age was 59.5 years. Out of 205 cases studied, 204 were of the black race. Only one patient was of the white race. The patients were referred to the gastroenterology service, coming from various national health units, considering that the MCH units were the ones who referred the most, with 89.8% (184/205).

The cancers located in the middle third were more frequent, with 48.8% (100/205), following the lower third with 29.8% (61/205) and the upper third with 21.5% (44/205). The majority of studied cancers were SCC, with 92.7% (190/ 205), followed by ADC with 4.9% (10/205). There were verified other histologic types, such as Kaposi´s sarcoma and the esophagus spindle cell, making 2.4% (5/205) (Table 1).

**Table 1:** demographics, endoscopic and histologic characteristics of the participants

Characteristics	N= 205	
	N	%
**Sex**		
Male	116	56.6
Female	89	43.4
**Age ( years)**		
≤ 30	3	1.5
31-40	22	10.7
41-50	35	17.1
51-60	69	33.7
61-70	48	23.4
71-80	20	9.8
>80	8	3.9
**Naturality**		
Maputo	109	53.2
Gaza	46	22.4
Inhambane	35	17.1
Manica	2	1.0
Sofala	3	1.5
Tete	5	2.0
Zambézia	3	1.5
Nampula	2	1.0
Niassa	1	0.5
**Endoscopic Characteristics**		
Upper third	44	21.5
Middle third	100	48.8
Lower third	61	29.8
**Histologic characteristics**		
SCC	190	92.7
ADC	10	4.9
Other	5	2.4

There was no relationship between histologic findings and the endoscopic location of cancer, with SCC occurring more frequently in the middle third (48.9%), and ADC occurring more regularly in the lower third (60%). Assuming a 95% confidence level, the relationship was not statistically significant with a Person's Chi-square of 0.124 (Table 2). Of the diagnosed cases, only 14 underwent surgical resection, being 10 early cancers and 4 locally advanced cancers.

**Table 2:** relation between histologic diagnosis and the endoscopic topography of cancer

		Endoscopic topography of cancer			Total	P
		Upper third	Middle third	Lower third		
**Histologic diagnosis**	ADC	0	4	6	10	**0.124**
	SCC	44	93	53	190	
	Other	0	3	2	5	
**Total**		44	100	61	205	

Abbreviation: ADC- Adenocarcinoma; SCC- Squamous cell carcinoma

There were 34 cases confirmed as being dead after the diagnosis up to the time the data was collected. The average lifespan after diagnosis in patients confirmed as death was 10.5 months.

## Discussion

This study with retrospective data has had the main objective to analyse the demographic, endoscopic and histologic profile of the patients with EC attended in the gastroenterology service of MCH as from 2016 to 2018.

Our study has noticed a significant frequency of cases in females, which contrasts with various epidemiological studies carried out in Africa [37]. However, it goes through the same line of a recent study in Mozambique that verified a major EC frequency in females [11]. This high frequency observed in females in Mozambique can be related to significant demand for health unities by women [38] and smoke inhalation resulting from the food preparation by using charcoal/ firewood in the environment with poor ventilation. These facts can contribute to exposing polycyclic aromatic hydrocarbons [39]. The most affected age group among our patients was between 51 to 60 years, being an average age of 59.5 years and a standard deviation of 12.9 years. This amount of standard deviation shows that there was a huge spread in age. The age found in this group goes in line with the recent studies carried out in Mozambique that shows the average age of 56.1 years and standard deviation of 13.2 [11]. Nevertheless, the studies in Africa show increased incidences in 40 years, registering the peak in 70 years [9]. This difference found in the studies in Mozambique and other countries in Africa can be due to reasons beyond being exposed to the smoke in places with no ventilation. Other factors include the ingestion of poorly conserved food, which leads to the production of mycotoxins (Aflatoxina B1 e Fumonisina B1), which is believed to be carcinogenic [40]. The majority of the patients in this study are from South of Mozambique, with 92.7% (190/205), of which Maputo adds up to 53.2% (109/205). The major record verified in the South, mainly in Maputo, can be justified because the study had been carried out in MCH, the country's major health unit with a huge capacity of endoscopic and histologic diagnosis. Objective conclusion cannot be extracted concerning EC frequency in other provinces. More studies should be carried out and for that in the hospitals that have endoscopic and pathology unit.

In relation to the race, 99.5% (204/205) were the black race, which is perfectly justified because the majority of Mozambicans are of the black race [41]. The most affected endoscopic location was the middle third with 48.8% (100/205), followed by the lower third with 29.8% (61/205) and the superior third with 21.5% (44/205). The cancer being more located in the middle third can be justified by the histologic type that is more frequent in the patients in the case studied, the SCC, which commonly affects the middle third, following the distal third of esophagus [19].

As it had been referred previously, the SCC was the most frequent with 92.7% (190/205), following the ADC with 4.9% (10/205). This tendency is in line with the epidemiologic standard of underdeveloped countries, where the risk factors for this type of cancer are more frequent [20]. Although there is a relation between histologic diagnosis and the endoscopic topography of cancer, this study cannot show this relation. The relation was not statistically significant, with a p=0.124, and can be justified by the fact that the sample is not being sufficiently representative.

Future studies should be conducted to describe the unknown causes in high prevalence regions. Additional genetic and genomic studies are needed, particularly in Africa, which is not well represented in the current genomic literature. Such studies can provide data for risk stratification and contribute to the understanding of etiological heterogeneity. The development of a clinically useful non-endoscopic test should be of high priority, as it can dramatically affect the load of SCC in high incidence regions [37].

**Limitations:** the constraints found in the study were: lack of information related to variables of the studies in the reports, lack of some clinical reports, caused by loss or damage, absence of some histologic reports, and being a hospital basis study, it does not present an actual dimension of the problem of the population.

## Conclusion

The main conclusions of this study are: the female was the most affected gender; the major number of cases in the patients of south zone 92.7%, being Maputo City and the province and together presented more cases; the most affected endoscopic local was the middle third; the most frequent histologic type was SCC.

### What is known about this topic

- Esophageal carcinoma is a very common gastrointestinal cancer in Mozambique;
- In our population, the most frequent histologic type is squamous cell carcinoma;
- In our population it is more frequent in females, contrary to the epidemiology of other countries.

### What this study adds

- It is one more study to demonstrate the high frequency of esophageal cancer in our country;
- Increases the level of alert to this public health problem and enforce policies to restrict risk factors that lead to an increase in SCC;
- Arouse curiosity in this topic and idealise investigation of further studies to characterise better the different risk factors linked to our population.

## References

[1] Guyton AC, Hall JE (2000). Guyton & Hall-Tratado de fisiologia medica. Rio de Janeiro-Guanabara Koogan.

[2] Lamb PJ, Griffin SM (2005). The Anatomy and Physiology of the Oesophagus the Oesophagus.

[3] Zhang Y (2013). Epidemiology of esophageal cancer. World J Gastroentero.

[4] Sung H, Ferlay J, Siegel RL, Laversanne M, Soerjomataram I, Jemal A, Global Bray F (2021). cancer statistics 2020: GLOBOCAN estimates of incidence and mortality worldwide for 36 cancers in 185 countries. CA Cancer J Clin.

[5] Napier KJ, Scheerer M, Misra S (2014). Esophageal cancer?: A Review of epidemiology pathogenesis staging workup and treatment modalities. World J Gastrointest Oncol.

[6] Pennathur A, Gibson MK, Jobe BA, Luketich JD (2013). Oesophageal carcinoma. Lancet.

[7] Kamangar F, Dores GM, Anderson W (2017). Patterns of Cancer Incidence Mortality and Prevalence Across Five Continents?: Defining Priorities to Reduce Cancer Disparities in Different Geographic Regions of the World. Journal of clinical oncology.

[8] Karamanou M, Markatos K, Papaioannou TG, Zografos G, Androutsos G (2017). Hallmarks in history of esophageal carcinoma. JBUON.

[9] Asombang AW, Chishinga N, Nkhoma A, Chipaila J, Nsokolo B, Manda-mapalo M (2019). Systematic review and meta-analysis of esophageal cancer in Africa: Epidemiology, risk factors, management and outcomes. World J Gastroentero.

[10] Lorenzoni CF, Ferro J, Carrilho C, Colombet M, Parkin DM (2020). Cancer in Mozambique: Results from two population-based cancer registries. Int J Cancer.

[11] Come J, Castro C, Morais A, Cossa M, Modcoicar P, Tulsidâs S (2018). Clinical and Pathologic Profiles of Esophageal Cancer in Mozambique: A Study of Consecutive Patients Admitted to Maputo Central Hospital. J Glob Oncol.

[12] Mao W, Zheng W, Ling Z (2011). MINI-REVIEW Epidemiologic Risk Factors for Esophageal Cancer Development. Asian Pacific Journal of Cancer Prevention.

[13] On A, Iva B (2014). Cancer of the esophagus?: histopathological sub-types in northern Uganda. African Health sciences.

[14] Wapnik S, Zanamwe LND, Chitiyo M, Mynors JM (1972). The Esophagus in Central Africa. CHEST.

[15] Raman R, Deorah S, Mcdowell BD (2010). Changing incidence of esophageal cancer among white women?: analysis of SEER data ( 1992-2010 ). Contemporary oncology.

[16] Hongo M, Nagasaki Y, Shoji T (2009). Epidemiology of esophageal cancer?: Orient to Occident. Effects of chronology geography and ethnicity. Journal of Gastroenterology and Hepatology.

[17] Cheng M, Zhang L, Borok M, Chokunonga E, Korir A, Wabinga HR (2016). The incidence of oesophageal cancer in Eastern Africa: Identification of a new geographic hot spot?. Cancer Epidemiol.

[18] Bassetti-soares E, Siqueira T, Delgado J (2009). Câncer de Esôfago?: Perfil das Manifestações Clínicas Histologia Localização e Comportamento Metastático em Pacientes Submetidos a Tratamento Oncológico em um Centro de Referência em Minas Gerais. Revista Brasileira de Cancerologia.

[19] Mchembe MD, Rambau PF, Chalya PL, Jaka H, Koy M, Mahalu W (2013). Endoscopic and clinicopathological patterns of esophageal cancer in Tanzania?: experiences from two tertiary health institutions. World J Surg Oncol.

[20] José M, Arnal D, Arenas ÁF Arbeloa ÁL (2015). Esophageal cancer?: Risk factors screening and endoscopic treatment in Western and Eastern countries. World J Gastroenterol.

[21] Huang F, Yu S (2018). Esophageal cancer?: Risk factors genetic association and treatment. Asian J Surg.

[22] Daly JM, Fry WA, Little AG, Winchester DP, McKee RF, Stewart AK, Fremgen A (2000). Esophageal cancer: Results of American College of Surgeons patient care evaluation study. J Am Coll Surg.

[23] Lordick F, Mariette C, Haustermans K, Arnold D, Committee G (2016). clinical practice guidelines Oesophageal cancer?: ESMO Clinical Practice Guidelines for diagnosis treatment and follow-up † clinical practice guidelines. Annals of Oncology.

[24] De Lange T, Halvorsen P, Riegler M (2018). Methodology to develop machine learning algorithms to improve performance in gastrointestinal endoscopy. World J Gastroenterol.

[25] Tustumi F, Kimura CMS, Takeda FR, Uema RH, Salum RAA, Ribeiro-Junior U (2016). Prognostic factors and survival analysis in esophageal carcinoma. ABCD Arq Bras Cir Dig.

[26] Rice TW, Patil DT, Blackstone EH (2017). 8^th^edition AJCC/UICC staging of cancers of the esophagus and esophagogastric junction: Application to clinical practice. Ann Cardiothorac Surg.

[27] De JE, De MAC, Pérez GCF, Pérez RR, Delgado AÁ (2016). Cáncer de esófago?: particularidades anatómicas estadificación y técnicas de imagen. Radiologia.

[28] Queiroga RC (2006). Esophageal Cancer?: Epidemiology Diagnosis and Treatment. Revista Brasileira de Cancerologia.

[29] Vining P, Birdas TJ (2019). Management of clinical T2N0 esophageal cancer?: a review. Journal of Thoracic Disease.

[30] Mayanagi S, Irino T, Kawakubo H, Kitagwa Y (2019). Neoadjuvant treatment strategy for locally advanced thoracic esophageal cancer. Ann Gastroenterol Surg.

[31] Kato H, Nakajima M (2013). Treatments for esophageal cancer?: a review. Gen Thorac Cardiovasc Surg.

[32] W?odarczyk J, Kuzdzal J (2018). Stenting in Palliation of Unresectable Esophageal Cancer. World J Surg.

[33] Chen H, Shen W, Liu K (2017). Radioactive self-expanding stents for palliative management of unresectable esophageal cancer?: a systematic review and meta-analysis. Diseases ofthe Esophagus.

[34] Hayes T, Riddell A (2017). Staging in Esophageal and Gastric Cancers. Hematol Oncol Clin N Am.

[35] Watanabe M, Otake R, Kozuki R, Toihata T, Takahashi K, Okamura A (2019). Recent progress in multidisciplinary treatment for patients with esophageal cancer. Surg Today.

[36] Trainini MM, Torres J da S (2015). Mídias Sociais como ferramentas de Estratégias de Marketing. Rev Ciência e Conhecimento.

[37] Middleton DRS, Bouaoun L, Hanisch R, Bray F, Dzamalala C, Chasimpha S (2018). Esophageal cancer male to female incidence ratios in Africa: A systematic review and meta-analysis of geographic, time and age trends. Cancer Epidemiol.

[38] Yeatman S, Chamberlin S, Dovel K (2018). Women´s (health) work: A population-based, cross-sectional study of gender differences in time spent seeking health care in Malawi. PLoS One.

[39] Titcombe ME, Simcik M (2011). Personal and indoor exposure to PM2.5 and polycyclic aromatic hydrocarbons in the southern highlands of Tanzania: A pilot-scale study. Environ Monit Assess.

[40] Come J, Cambaza E, Ferreira R, Da Costa JMC, Carrilho C, Santos LL (2019). Esophageal cancer in Mozambique?: should mycotoxins be a concern?. PAJM.

[41] INE (2019). Quarto recenciamento geral da população e habitação 2017 resultados definitivos - Moçambique. Instituto nacional de estatistica.

